# The Influence of the Disadvantaged Mindset on System-Justifying Beliefs

**DOI:** 10.3389/fpsyg.2021.787417

**Published:** 2022-02-03

**Authors:** Lihua Yang, Shujun Tang, Kai Li

**Affiliations:** ^1^School of Foreign Languages, Jiangxi Normal University, Nanchang, China; ^2^School of Marxism, Jiangxi Normal University, Nanchang, China; ^3^School of Psychology, Jiangxi Normal University, Nanchang, China

**Keywords:** disadvantaged mindset, theory of system justification, collectivism, disadvantaged groups, social system

## Abstract

System justification theory holds that disadvantaged groups rationalize the current social system, even if it is unfavorable to them. Epistemic, relational, and existential needs are factors that explain this phenomenon. However, the literature has not yet examined and explained when disadvantaged groups no longer rationalize current social systems. This study uses a questionnaire survey method (*N* = 745) to study the moderating effect of collectivism on disadvantaged mindset and system-justifying beliefs. It found that collectivism can influence the predictive effect of disadvantaged mindset on system-justifying beliefs. For people who scored low in collectivism, a disadvantaged mindset can significantly negatively predict system-justifying beliefs; for those who scored high in collectivism, a disadvantaged mindset no longer predicts system-justifying beliefs. Therefore, these results show that collectivist values are important for explaining system justification in disadvantaged groups. When collectivist values decline, the level of rationalization of the social system by disadvantaged groups also decreases.

## Introduction

That sometimes low-status, disadvantaged groups rationalize and defend existing social systems that are mostly unfavorable to them is paradoxical. Many studies have found that individuals tend to perceive unreasonable social systems as reasonable (Jost et al., 2012). For example, people with low incomes often oppose income redistribution (Kluegel and Smith, 1986), and women accept gender stereotypes and traditional gender roles (e.g., Glick et al., 2000). Low-income respondents and African Americans were more likely than others to support limitations to the rights of citizens and media representatives to criticize the government (Jost et al., 2003). To explain this phenomenon, scholars have formulated the system justification theory (Henry and Saul, 2006). According to this theory, people rationalize a system due to epistemic, relational, and existential (Jost and Banaji, 1994; van der Toorn and Jost, 2014; Jost, 2020). Despite the fact they dislike the situation initially, people will consciously or unconsciously convince themselves that the current social system is reasonable, or even selectively ignore some of its shortcomings (Blasi and Jost, 2006).

However, although some research evidence has been obtained to support of this theory, many studies have also found the opposite: disadvantaged groups are more dissatisfied with the social system (Yang et al., 2019). The circumstances under which people in disadvantaged positions no longer rationalize a system that is unfavorable to them are worthy of discussion (Owuamalam et al., 2019). The current study aims to explore the relationship between the mindset of disadvantaged individuals and system-justification beliefs.

According to system justification theory, in addition to defending themselves and their group’s interests, people tend to defend and rationalize the *status quo* (including the current social, economic, and political systems), sometimes at the expense of some of their own interests (Jost et al., 2004). According to this view, system justification is natural because people are psychologically motivated to rationalize the system (Jost, 2019). Disadvantaged people are unable to change their situation, so they employ a simple strategy of rationalizing the system to maintain cognitive balance. If they think that the system is unreasonable, they will experience cognitive dissonance; therefore, to maintain cognitive coordination, they are motivated to believe in the rationality of the system (Jost, 2017).

A disadvantaged mindset establishes itself when disadvantaged people recognize a painful reality for themselves (when compared to others). It is the individual’s recognition of their strengths and weaknesses in competing to obtain a better life or higher social status than others (Li, 2020). Furthermore, it refers to the psychological tendency of individuals who usually feel disadvantaged in society, including feeling that their life is more difficult (Kuper and Kuper, 2001). Some research studies have found that system justification was positively associated with socioeconomic status (e.g., Lönnqvist et al., 2021). Compared to individuals with higher incomes, disadvantaged individuals feel that the system cannot meet their needs and that they are unable to change it. This evidence suggests that a disadvantaged mindset is negatively associated with system justification.

Collectivism places more importance on the group and society than on an individual’s objectives (Oyserman et al., 2002). It can moderate the relationship between a disadvantaged mindset and system-justifying beliefs. On the one hand, individualism and collectivism are cultural dimensions that measure “the degree of interdependence a society maintains among individuals” (Hofstede, 1984, p. 83). Thus, the individualism/collectivism is a moderator of the relationship between use of e-learning systems and individual performance (Aparicio et al., 2016). On the other hand, to avoid social exclusion, people may be motivated to share their perception of reality with high system-justifiers (Cheung et al., 2011). These relational needs increase system-justification tendencies (Hess and Ledgerwood, 2014); thus, collectivism may strengthen the influence of a disadvantaged mindset on system-justification beliefs.

The present study aims to investigate the effects of a disadvantaged mindset on system-justifying beliefs, and the potential moderating role of collectivism. It uses a questionnaire survey method to study the moderating role of collectivism. We developed the following research hypotheses for our study:

H1: For people who scored low in collectivism, a disadvantaged mindset can significantly negatively predict system-justifying beliefs.

H2: For people who scored high in collectivism, a disadvantaged mindset does not predict system-justifying beliefs.

## Materials and Methods

### Research Participants

G*Power (Faul et al., 2007) was used to conduct a power analysis with alpha = 0.05, power = 0.95, and a small effect size for a regression analysis of three predictors on a continuous outcome. To attain a small effect (*f*^2^ = 0.02) with those parameters, 652 participants were needed. A total of 745 students (23.8% male, 74.8% female, 1.5% missing) at a university in Jiangxi Province participated in this study. Participants were 20.56 years of age on average (SD = 1.76).

### Assessment Tools and Experimental Material

The following assessment tools and experimental material were used in the current research:

(i) The disadvantaged mindset scale (Li, 2020), which consists of eight items (e.g., “Even if I work hard, it is difficult to cope with the potential risks in life,” “Compared with most people, I work harder but I am not more successful”), was used in this study. The evaluation was conducted using a seven-point scale (1 = completely disagree; 7 = completely agree), where higher scores indicated greater disadvantaged mindset. Cronbach’s α for this scale was 0.78.
(ii) The scale of system justification (Kay and Jost, 2003), which consists of eight items [e.g., “In general, I find society to be fair,” “Our current society needs to be radically restructured(R)”], was used in this study. The items were evaluated on a seven-point scale (1 = “completely disagree”; 7 = “completely agree”), where higher scores indicated stronger system-justifying beliefs. Cronbach’s α for this scale was 0.82.
(iii) The scale of collectivism (Yoo and Donthu, 2005), which consists of six items (e.g., “Individuals should sacrifice self-interest for the group that they belong to,” “Individuals should pursue their goals after considering the welfare of the group”), was used in this study. The items were evaluated on a seven-point scale (1 = “completely disagree”; 7 = “completely agree”). In this scale, higher scores indicated greater collectivistic tendencies. Cronbach’s α for this study was 0.89.

### Experimental Procedure

All procedures in this study were performed in accordance with the standards of the Ethics Board of Jiangxi Normal University. All participants provided written informed consent before they participated in this study.

### Statistical Analysis

Given that all the data were collected *via* questionnaire, we adopted the recommendations of MacKenzie and Podsakoff (2012) to control for the common potential biases in this method. Specifically, the data collection process was entirely anonymous, and after the data collection had been completed, we used Harman’s single factor test to identify the potential level of common method variance. The results indicated that six factors had eigenvalues greater than 1, and the variance of the first factor was 24.59% (i.e., less than 40%, which is the lowest threshold commonly used for Harman’s test).

## Results

### Results of Descriptive Statistics

As shown in Table 1, disadvantaged mindset and system-justifying beliefs were significantly negatively correlated (*r* = −0.16, *p* < 0.01), thus supporting Hypothesis 1. In addition, a significant positive correlation between collectivism and system-justifying beliefs (*r* = 0.38, *p* < 0.001) and a significant negative correlation between a disadvantaged mindset and collectivism (*r* = −0.12, *p* < 0.01) were found.

**TABLE 1:** Descriptive statistics and intercorrelations between variables (*n* = 745).

	Mean	SD	1	2	3	4	5
(1) Gender	0.24	0.43	1				
(2) Age	20.55	1.76	−0.02	1			
(3) Disadvantaged mindset	3.80	0.87	0.001	0.01	1		
(4) Collectivism	4.70	1.19	−0.07	0.07	−0.12**	1	
(5) SJB	4.79	0.60	−0.05	−0.08*	−0.16**	0.38***	1

SJB, system-justifying belief; For gender, 0 = female, 1 = male. *p < 0.05, **p < 0.01, and ***p < 0.001.

### Hypotheses Testing

To examine the effects of a disadvantaged mindset and collectivism on system-justifying beliefs, we conducted a hierarchical regression analysis. First, gender and age were entered into the regression equation; second, disadvantaged mindset and collectivism were entered into the regression equation as predictors of system-justifying belief; third, the interaction term (disadvantaged mindset × collectivism) was entered. The results of this analysis are shown in Table 2.

**TABLE 2:** Hierarchical multiple regression on system-justifying belief (n = 745).

	Step 1			Step 2			Step 3		
Variables	*B*	SE	95% CI	*B*	SE	95% CI	*B*	SE	95% CI
Age	−0.03	0.01	[−0.05, −0.01]	−0.03	0.01	[−0.05, −0.01]	−0.03	0.01	[−0.05, −0.001]
Gender	−0.07	0.05	[−0.17, 0.04]	−0.03	0.05	[−0.13, 0.06]	−0.04	0.05	[−0.13, 0.06]
Disadvantaged mindset				−0.07	0.02	[−0.12, −0.03]	−0.33	0.09	[−0.50, −0.16]
Collectivism				0.19	0.02	[0.16, 0.22]	−0.01	0.07	[−0.15, 0.12]
Interaction							0.06	0.02	[0.02, 0.09]
*R* ^2^	0.09			0.41			0.43		
*F*	2.81			35.79***			30.80***		

For gender, 0 = female, 1 = male. *p < 0.05, **p < 0.01, and ***p < 0.001.

Simple slope tests revealed that individuals with low collectivism (−1 SD) demonstrated a significant negative relationship between a disadvantaged mindset and system-justifying beliefs (*B* = −0.14, SE = 0.03, *t* = −4.51, *p* < 0.001, 95%CI = [−0.20, −0.08]). In contrast, individuals with high collectivism (+1 SD) did not display any significant relationship between disadvantaged mindset and system-justifying beliefs (*B* = −0.004, SE = 0.03, *t* = −0.12, *p* = 0.90, 95%CI = [−0.07, 0.06]).

## Discussion

System justification theory emphasizes that system justification is essentially a motivational and goal-oriented psychological process (Jost, 2019), and that such beliefs are related to individual motivational social cognition (Wojcik et al., 2015). The present study found that a disadvantaged mindset is negatively correlated with system justification when collectivism is low, consistent with related research studies (e.g., Kraus and Callaghan, 2014; Kraus and Tan, 2015). In contrast, for people with high collectivism, a disadvantaged mindset does not predict system-justification beliefs. Our research broadens the scientific understanding of relationship between disadvantaged mindset and system-justification beliefs.

First, collectivism moderates the correlation between disadvantaged mindset and system-justification beliefs. This finding effectively explains some contradictory findings in the system justification theory literature describing how lower status groups will engage in more system justification than higher status groups. Collectivists are most likely to engage in self-enhancement along collectivist rather than individualist dimensions. Lower collectivists not inclined to rationalize systems (Sedikides et al., 2003). Thus, when people display high levels of collectivism, their system-justification beliefs do not decrease although they have expended substantial effort yet have not been rewarded. This finding supports the hypothesis of Owuamalam et al. (2019) specifically that a greater sense of in-group membership among collectivists that leads them work harder to justify the system. In contrast, individualists would be less likely to identify with a collective system when they expend effort yet are not rewarded. Overall, the relationships between a disadvantaged mindset, system-justifying beliefs, and collectivism should be more thoroughly explored in future studies.

Second, the disadvantaged mindset construct can help us better understand system justification theory. The disadvantaged mindset emphasizes how people’s recognition that an individual has worked hard yet has not been rewarded (Li, 2020), if they rationalize such systems, is influenced by other factors (such as collectivism and individualism). When individuals display low levels of collectivism, there was a significant negative relationship between a disadvantaged mindset and system-justifying beliefs. When they have strong collectivistic beliefs, a less disadvantaged mindset does not decrease the level of system-justifying belief. The negative correlation of disadvantaged mindset and system-justification belief is weak, which suggests that the disadvantaged mindset is not a mere inversion of system justification. Thus, when people are high in disadvantaged mindset and high in system-justification beliefs, they might think that they are exceptionally unfortunate or incompetent, which may have deleterious effects on their mental health.

Third, this study has some limitations. Our results were obtained from a correlational analysis, therefore it cannot be determined whether disadvantaged mindset leads to lower collectivism and higher individualism, or if the relationship works in the inverse direction, or all factors are related to another unknown or unrecognized factor. Future research may benefit from exploring these relationships using controlled experiments conducted in the laboratory, or a moderation analysis. Future studies might examine people’s early childhood environments to analyze how the disadvantaged mindset influences collectivism, individualism, and system-justifying beliefs.

## Conclusion

Collectivism can influence the predictive effect of a disadvantaged mindset on system-justifying beliefs. For people scoring low in collectivism, the disadvantaged mindset significantly negatively predicts system-justifying beliefs; for those who scored high in collectivism, the disadvantaged mindset does not predict system-justifying beliefs. The results of this study show that collectivist values are important for explaining system justification in disadvantaged groups. With a decline in collectivist values, rationalization of the social system by disadvantaged groups also decreases.

## Data Availability Statement

The original contributions presented in the study are included in the article/supplementary material, further inquiries can be directed to the corresponding author.

## Ethics Statement

The studies involving human participants were reviewed and approved by the Ethics Board of Jiangxi Normal University. Written informed consent to participate in this study was provided by the participants’ legal guardian/next of kin.

## Author Contributions

LY developed and validated the theoretical model. ST contributed to the data preparation and analysis and manuscript writing. KL expanded the theoretical model and manuscript writing. All authors wrote the manuscript and approved the final version of the manuscript for submission.

## Conflict of Interest

The authors declare that the research was conducted in the absence of any commercial or financial relationships that could be construed as a potential conflict of interest.

## Publisher’s Note

All claims expressed in this article are solely those of the authors and do not necessarily represent those of their affiliated organizations, or those of the publisher, the editors and the reviewers. Any product that may be evaluated in this article, or claim that may be made by its manufacturer, is not guaranteed or endorsed by the publisher.
